# Fluorinated Carbohydrates as Lectin Ligands: ^19^F-Based Direct STD Monitoring for Detection of Anomeric Selectivity

**DOI:** 10.3390/biom5043177

**Published:** 2015-11-13

**Authors:** João P. Ribeiro, Tammo Diercks, Jesús Jiménez-Barbero, Sabine André, Hans-Joachim Gabius, Francisco Javier Cañada

**Affiliations:** 1 Centre de Recherches sur les Macromolécules Végétales, UPR5301, CNRS-Université Grenoble Alpes, BP53, 38041 Grenoble cédex 09, France; E-Mail: ribeiro@cermav.cnrs.fr; 2 CIC bioGUNE, Bizkaia Technological Park, Building 800, 48160 Derio, Spain; E-Mail: tdiercks@cicbiogune.es; 3 Ikerbasque, Basque Foundation for Science, 48013 Bilbao, Spain; 4 Institut für Physiologische Chemie, Tierärztliche Fakultät der Ludwig-Maximilians-Universität, Veterinärstr. 13, 80539 München, Germany; E-Mail: S.Andre@tiph.vetmed.uni-muenchen.de; 5 Chemical and Physical Biology, Centro de Investigaciones Biológicas, CIB-CSIC, Ramiro de Maeztu 9, 28040 Madrid, Spain

**Keywords:** agglutinin, carbohydrates, drug design, glycans, glycoproteins, lectins, sugar receptors

## Abstract

The characterization of the binding of reducing carbohydrates present as mixtures of anomers in solution to a sugar recepor (lectin) poses severe difficulties. In this situation, NMR spectroscopy enables the observation of signals for each anomer in the mixture by applying approaches based on ligand observation. Saturation transfer difference (STD) NMR allows fast and efficient screening of compound mixtures for reactivity to a receptor. Owing to the exceptionally favorable properties of ^19^F in NMR spectroscopy and the often complex ^1^H spectra of carbohydrates, ^19^F-containing sugars have the potential to be turned into versatile sensors for recognition. Extending the recently established ^1^H → ^1^H STDre^19^F-NMR technique, we here demonstrate its applicability to measure anomeric selectivity of binding in a model system using the plant lectin concanavalin A (ConA) and 2-deoxy-2-fluoro-d-mannose. Indeed, it is also possible to account for the mutual inhibition between the anomers on binding to the lectin by means of a kinetic model. The monitoring of ^19^F-NMR signal perturbation disclosed the relative activities of the anomers in solution and thus enabled the calculation of their binding affinity towards ConA. The obtained data show a preference for the α anomer that increases with temperature. This experimental approach can be extended to others systems of biomedical interest by testing human lectins with suitably tailored glycan derivatives.

## 1. Introduction

Glycoconjugates are abundant and ubiquitous in nature, suggesting functional significance. The enormous complexity of the glycan part of cellular glycoconjugates has delayed the interest in studying these biomolecules so that structure-activity relationships are emerging far behind those for proteins and nucleic acids [1]. In direct comparison, using sugars (the third alphabet of life) as platform, an unsurpassed density of information coding is realized in these glycan chains, the basis of the concept of the sugar code [2]. Strategically combined with sophisticated purification protocols and other analytical techniques, high-resolution ^1^H-nuclear magnetic resonance spectroscopy (NMR) has become a powerful tool in the quest to accomplish full structural elucidation of these natural “code words” [3,4,5,6]. For example, reporter-group signals provide essential information on the primary structure and substitution patterns [4,5,6]. When distinct glycan determinants are bound by their receptor (lectin) to “read the encoded information”, NMR continues to be a rich source of information on structural aspects, here on intimate details of the complex [7,8]. As a consequence of the broad physiological significance of glycan-lectin recognition processes, their study has revealed a potential for drug design [2]. Given the relevance of advances in NMR application for drug design [9,10], basic and applied research in structural glycobiology is sure to benefit from this technology. In this respect, monitoring different nuclei broadened in scope. Moving beyond ^1^H, ^13^C and ^15^N, the use of ^19^F was introduced four decades ago into the study of glycan-protein interactions, examining chemical shift perturbations and line broadening in the cases of lysozyme [11,12], wheat germ agglutinin [13,14] or concanavalin A [15]. Of note, fluorine has very favorable properties when considering NMR: the ^19^F nucleus, with 100% natural abundance, nuclear spin ½ and a gyromagnetic ratio close to that of ^1^H (0.94 γ_19F_/γ_1H_), has similar NMR sensitivity as a proton. Due to these favorable properties ^19^F-based NMR spectroscopy continues to open doors to new applications [16,17,18,19,20]. An additional benefit of using ^19^F-NMR is the absence of dynamic range and chemical exchange problems related to the large NMR signal of the common solvent water. In NMR studies of glycans, the residual water signal often masks key signals from the very diluted biological samples. The broad ^19^F-NMR observation range (200 ppm) can also avoid the frequent signal overlap in glycan ^1^H-NMR (less than 3 ppms wide for common carbohydrate molecules). This problem is further aggravated when working with mixtures of compounds. Overall, its pharmacological and NMR properties thus make the fluorine nucleus to an excellent atomic probe in interaction studies [16,17,18,19,20], prompting us to explore its use in protocols developed for drug screening, here adapted for a lectin (for recent reviews on biomedical aspects of glycan structure and recognition, please see [2,21,22,23]). Having started by using fluoro derivatives for probing into requirements for hydrogen binding [24,25,26,27] following classical chemical mapping strategies [28], it was thus tempting to proceed to evaluate ^19^F-NMR as reporter for lectin recognition. We here address the question on its applicability to infer anomer selectivity.

**Scheme 1:**
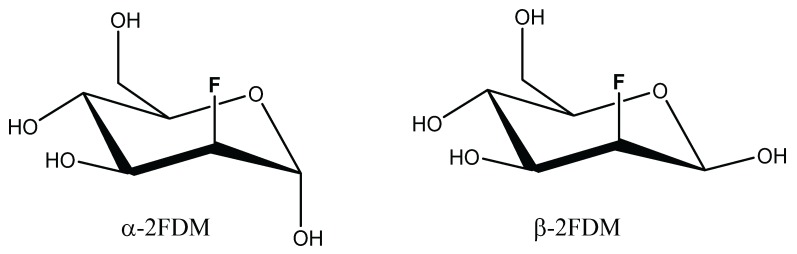
Formulas of 2-Deoxy-2-fluoro-d-mannose anomers.

The legume lectin concanavalin A (ConA), isolated from jack bean *(Canavalia ensiformis)* seeds, is a common test model [29]. It selectively binds mannose and glucose. Studies with simple sugars revealed that the OH groups at C-3, C-4 and C-6 positions are essential and the α-anomeric position is preferred [30,31]. Reflecting the specificity for mannose/glucose, the substituent and stereochemistry at the C-2 position is of minor importance. Thus, methyl α-glycosides [32] and their respective fluoro and deoxy derivatives [33] of these sugars are ligands of ConA. 2-Deoxy-2-fluoro-d-mannose (2FDM, Scheme 1), where the axial hydroxyl at position 2 is replaced by a fluorine atom, belongs to this group. In solution, 2FDM is present as a mixture of anomers, establishing an attractive test system for ^19^F-NMR to reveal anomer selectivity by a lectin.

Anomeric selectivity has been studied in enzymes involved in carbohydrate metabolism using kinetic approaches [34,35]. However, monitoring of substrate processing is not possible in the case of lectins, which by definition are devoid of any enzymatic activity on the bound ligand [36]. The common strategy to measure anomer selectivity engages a chemical modification at the anomeric position (a simple aglycon (methyl or isopropyl) introduction), hereby locking the molecule in its α or β configuration. Direct detection of differential recognition of each anomer without derivatives formation still remains a challenge, the available biophysical techniques in solution that relay information by receptor-based analysis provide an averaged macroscopic view of the recognition event involving the mixture of anomers in equilibrium. In principle, ^1^H-NMR spectroscopy may well pick up characteristic signals for each anomer, despite a chemical equilibrium exchange [37]. In addition to ^1^H, other nuclei can be used to determine the position of the anomeric equilibrium. As an instructive example, ^13^C-NMR has been recently used qualitatively to reveal the β-anomer preference for bacterial sugar-binding proteins involved in bacterial chemotaxis [38]. Moreover, a rare case of ^3^H-NMR was instrumental in detecting the anomeric preference of a maltose-binding protein [39]. As for ^19^F-NMR, it has been applied to monitor fluoroglucose exchange across membranes [40] as well as to differentiate the binding of *N*-trifluoroacetyl-glucosamine anomers to ConA [15] and more recently the binding of *N*-trifluoroacetyl-galactosamine anomers to winged bean agglutinin [41] following line broadening perturbations. With the aim to advance the status of ^19^F as versatile sensor in lectin research, we recently developed a ^19^F-detected STD NMR methodology, successfully tested for ConA [42,43]. Building on this foundation, the given strategy, in addition to overcome signal-overlap problems, can help to obtain quantitative information on the binding of each anomer by simplifying the observation to single and well-resolved fluorine signals for each of them. Obviously, such a procedure will circumvent the need of any chemical fixation of the anomeric position on the glycan.

STD NMR is a powerful and efficient technique that allows detecting association of low-molecular-weight ligands (<2000 Da) to comparatively large receptors (>20,000 Da) such as proteins [44,45]. Besides the identification of the ligand epitope (contact) STD can be used to measure dissociation constants by titrations experiments performed at different ligand concentrations [45,46]. However, as the transfer of spin saturation from the irradiated protein to the ligand is dependent on the irradiation time, the correlation between STD signals and the concentration of loaded binding sites cannot be obtained directly from the intensity of STD signals at a single saturation time. Thus, courses of saturation time dependency must be used to infer the initial velocities of saturation [47]. Also, the relaxation of ligand-saturated spins during the irradiation time, when the ligand is not bound to the protein, should also be taken into account [48,49,50]. Recently, we have applied a kinetic model based on the DynaFit software [51] that explicitly includes the rebinding and relaxation of already saturated ligand in one analysis of the experimental STD values [52]. Herein, we present the extension of this kinetic model to include, in an individualized way, the presence of the α and β anomers as two competing ligands in equilibrium.

## 2. Material and Methods

### 2.1. Chemicals and Reagents

2FDM was obtained from Toronto Research Chemicals (Toronto, ON, Canada).

### 2.2. Purification of ConA

The lectin was purified from extracts of of *Canavalia ensiformis* by affinity chromatography on mannose-bearing Sepharose 4B, as described previously [31].

### 2.3. NMR Experiments

The experiments were performed at 280, 290, 300 and 310K using New Era 5 mm borosilicate tubes (reference NE-SL5-7). Samples contained 90 µM ConA and 2FDM concentrations ranging from 0 to 12.1 mM, dissolved in 99.9% D_2_O-based PBS ([NaCl] = 138 mM; [KCl] = 2.7 mM; [Na_2_HPO_4_] = 10.1 mM; [NaH_2_PO_4_] = 1.8 mM) at pD 7.0.

STD experiments were carried out on a Bruker Advance III 600 MHz spectrometer equipped with a ^19^F, ^1^H double resonance fluorine-selective (SEF) probe optimized for direct ^19^F detection and equipped with a z-spoil gradient coil (Bruker Biospin, Rheinstetten, Germany). The experiments were recorded using the described ^1^H → ^1^H STDre^19^F-NMR pulse sequence [42]. Selective saturation of the protein signal was obtained using a standard STD pulse train of multiple Gaussian pulses with a saturation time ranging between 0.75 and 12 s depending of the experiment. *On-*resonance irradiation of the protein was performed at a proton chemical shift of 0.93 ppm, and *off*-resonance irradiation was set to 145 ppm. STD spectra were acquired with a total of transients varying from 128 to 1 K.

Inversion recovery experiments to measure T1 relaxation times were carried out on samples with 2FDM at 12.1 mM and by means of a pseudo 2D spectrum recording a series of 32 fluorine spin inversion spectra with proton decoupling, varying the recovery delay time after spin inversion between 0.75 s up to 8.5 s and a prescan delay of 4 s. All fluorine spectra were recorded using a 40 ppm spectral width centered at −210 ppm (trifluoroacetic acid signal at −76.55 ppm was used as fluorine reference). Spectra were processed with TOPSPIN2.1 (Bruker, Rheinstetten, Germany).

### 2.4. Kinetic Model

The program DynaFit [51] was used to process the experimental data within the model. As follows, we describe the components, chemical equations (Equations 1–17), kinetic constants, concentrations and experimental data sets (Equations 18 and 19) that were used:

#### 2.4.1. Components

Ligand, 2FDM; [α]_total_ + [α]_total_ = [2FDM]_total_

R, receptor (ConA); R*, steady-state saturated receptor during *on*-resonance irradiation time

α, unsaturated α anomer; β, unsaturated β anomer

α*, saturated α anomer; β*, saturated β anomer

#### 2.4.2. Equations

R* + α ⇆ R*α*k_off_*_α_/*k_on_* = K_Dα_ (*k_on_*_α_ ≡ *k_on_*_β_ ≡ *k_on_*)(1)R* + β ⇆ R*β*k_off_*_β_/*k_on_* = K_Dβ_ (*k_on_*_β_ ≡ *k_on_*_α_ ≡ *k_on_*)(2)R*α → R*α**k_m_* (*k_m_*_α_ ≡ *k_m_*_β_)(3)R*β → R*β**k_m_* (*k_m_*_β_ ≡ *k_m_*_α_)(4)α* → α*k_r_*_α_(5)β* → β*k_r_*_β_(6)R* + α* ⇆ R*α**k_off_*_α_/*k_on_* (K_Dα*_ ≡ K_Dα_)(7)R* + β* ⇆ R*β**k_off_*_β_/*k_on_* (K_Dβ*_ ≡ K_Dβ_)(8)

#### 2.4.3. Derived Differential Equations Used in Dynafit Fitting Proccedure

d[R*]/dt = −*k_on_*[R*][α] + *k_off_*_α_[R*α] − *k_on_*[R*][α*] + *k_off_*_α_[R*α*] − *k_on_*[R*][β] + *k_off_*_β_[R*β] − *k_on_*[R*][β*] + *k_off_*_β_[R*β*](9)d[α]/dt = −*k_on_*[R*][α] + *k_off_*_α_[R*α] + *k_r_*_α_[α*](10)d[α*]/dt = −*k_on_*[R*][α*] + *k_off_*_α_[R*α*] − *k_r_*_α_[α*](11)d[β]/dt = −*k_on_*[R*][β] + *k_off_*_β_[R*β] + *k_r_*_β_[β*](12)d[β*]/dt = −*k_on_*[R*][β*] + *k_off_*_β_[R*β*] − *k_r_*_β_[β*](13)d[R*α]/dt = +*k_on_*[R*][α] − *k_off_*_α_[R*α] − *k_m_*[R*α](14)d[R*β]/dt = +*k_on_*[R*][β] − *k_off_*_β_[R*β] − *k_m_*[R*β](15)d[R*α*]/dt = +*k_on_*[R*][α*] − *k_off_*_α_[R*α*] + *k_m_*[R*α](16)d[R*β*]/dt = +*k_on_* [R*][β*] − *k_off_*_β_[R*β*] + *k_m_*[R*β](17)

#### 2.4.4. Constants

*k_on_*, fixed constant at 10^8^ M^−1^ s^−1^ for both anomers (approximation to diffusion-controlled process).

*k_off_*_α_; *k_off_*_β_; *k_m_*; *k_r_*_α_; *k_r_*_β_. Adjustable constants.

#### 2.4.5. Concentrations

Concentration of ConA (R*) was 0.09 mM in all experiments: [α]_total_ and [β]_total_ were calculated by knowing the total concentration of 2FDM and the anomeric ratio, c_anomer_, obtained from the integration of the corresponding fluorine NMR peaks:

[α]_total_ + [β]_total_ = [2FDM]_total_; concentration used: 0.96; 1.94; 4.14; 5.57 and 12.1 mM

c_anomer_ = [α]_total_/[β]_total_, constant value for each temperature: (1.92; 1.92; 1.82; 1.72 for 280 K, 290 K, 300 K and 310 K respectively).

#### 2.4.6. Experimental Data Sets

For every temperature (280 K, 290 K, 300 K and 310 K) and 2FDM concentration (0.96; 1.94; 4.14; 5.57 and 12.1 mM) the corresponding saturation time course (0.75, 1.5, 3, 6 and 12 s) data series of formal concentrations of saturated anomers, α* and β* were obtained from the experimental percentages of saturation of each anomer:

α* = %STDα × [α]_total_ (18)

β* = %STDβ × [β]_total_ (19)

## 3. Results and Discussions

**Figure 1:**
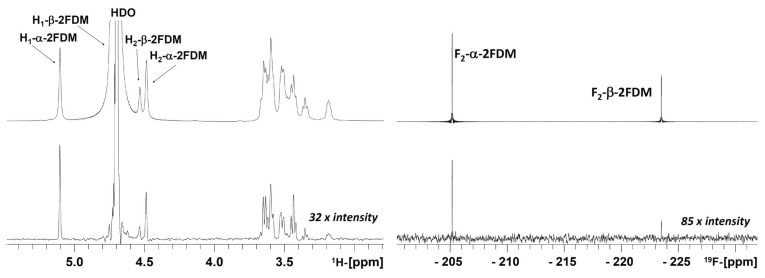
^1^H → ^1^H STD and ^1^H → ^1^H STDre^19^F STD decoupled spectra of a 2FDM/ConA sample, in 99% D_2_O at 280 K and 0.75 s irradiation. At the left: ^1^H → ^1^H STD spectrum (bottom) and corresponding *off*-resonance spectrum (at the top). Several proton signals, H_1_ and H_2_ of β-2FDM and H_2_ of α-2FDM, are positioned under or very close to the HDO signal at this temperature and its STD information is lost; at the right: ^1^H → ^1^H STDre^19^F (bottom) and the reference ^19^F *off*-resonance spectrum (at the top). Ligand (2FDM) and lectin (ConA) were present at 12.1 mM and 90 µM concentrations, respectively.

As mentioned in the introduction, the ^19^F-observed STD NMR approach does not suffer from signal overlap when dealing with carbohydrates. Thus, it yields a maximally simplified, background-free spectrum of α and β anomers. In this system, the regular ^1^H → ^1^H STD analysis would provide incomplete information, due to the overlap of proton signals (either H_1_ or H_2_ of the ligands) with the signal of residual HDO (Figure 1, left).

The prerequisite for this type of analysis is the existence of a significant difference between the relative STD signal (ratio of signal in the difference spectra and signal in the *off*-resonance spectra) of the α and the β anomer, which is fulfilled (Figure 1, right). This clearly observable difference between both anomers (1.1% and 0.5% STD for α and β, respectively at 280 K and with 0.75 s irradiation) makes it possible to quantify the binding affinity of each anomer.

First, a control experiment was conducted with 2FDM in the absence and presence of ConA, with the purpose of testing if any direct saturation transfer between the anomers occurs due to the anomeric exchange. This blank experiment corresponds to the regular saturation transfer experiment (as employed to detect dimethyl formamide chemical exchange around the amide bond). The irradiation on α-2FDM, with saturation times (t_sat_) ranging from 0.75 to 60 s, yielded no β-2FDM ^19^F signal (and *vice versa*), indicating that, at the time scale of the STD scan acquisitions, the kinetics of the anomeric exchange are considerably slower and do not interfere with the kinetics of the intermolecular (protein-sugar) saturation transfer. Therefore, the behavior of each anomer can be independently evaluated.

**Figure 2:**
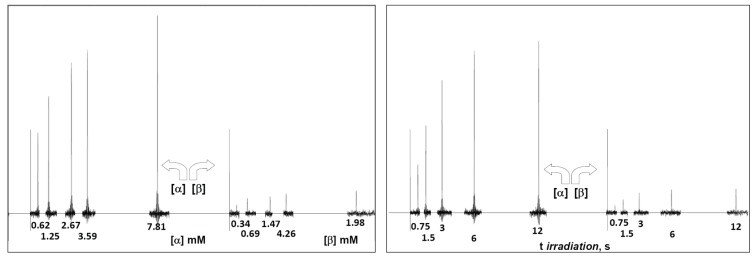
(**Left**) Serial STD spectra as a function of ligand concentration obtained at 300 K, 0.09 mM ConA and 3 s saturation time with anomeric percentages of 64.5% and 35.5% for α-2FDM and β-2FDM, respectively. The concentration of each anomer is below each signal, and each spectrum is shifted proportionally to the variable condition (ligand concentration); (**Right**) Serial STD difference spectra as a function of saturation time obtained at 290 K with 0.09 mM ConA and 12.1 mM total concentration of 2DFM with 65.8% and 34.2% for α and β anomer, respectively; the saturation time of each experiment is below each signal, and each spectrum is shifted proportionally to the variable condition (spin irradiation time).

**Figure 3:**
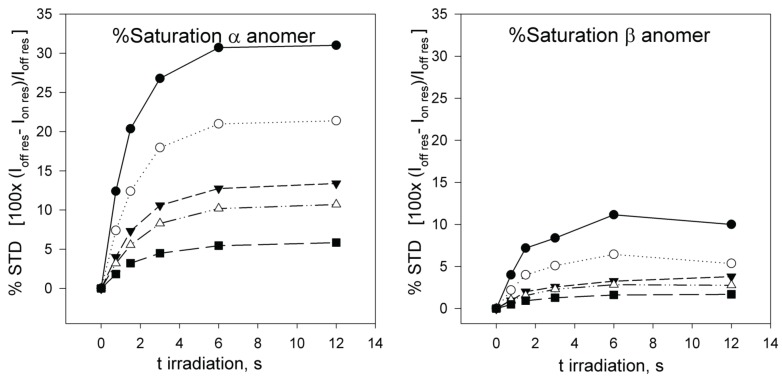
% STD build-up curves obtained at 290 K for α-2FDM (**left**) and β-2FDM (**right**) in the presence of ConA (90 µM) at several total concentrations of monosaccharide 0.96 (●); 1.94 (○); 4.14 (▼); 5.57 (Δ); and 12.1 mM (■) (anomer percentages 66% α-2FDM and 34% β-2FDM). The representation of ^19^F STD intensity *versus* saturation time shows that a stationary state, where saturation is compensated by relaxation, is reached at an approximate spin irradiation time of 6 s.

Next, full-scale saturation curves at different ligand concentrations were obtained by performing series of STD experiments with increasing saturation times (0.75, 1.5, 3, 6 and 12 s) and at four different temperatures (280, 290, 300 and 310 K). As examples of STD spectra, Figure 2 shows the raw data of a set of overlaid STD experiments. In one set, the STD intensity dependency on sugar concentration at one protein saturation time (3 s) and one fixed temperature (300 K) is shown. The alternative set displays the STD intensity dependency on the saturation time at one particular sugar concentration (12.1 mM) and a different temperature (290 K). At each temperature, the anomeric ratio was calculated from the intensity ratio of the fluorine signals between both anomers. The α/β ratio varies between 1.7 (310 K) and 1.9 (280 K) in favor of α anomer, as expected for a manno- configuration, and is close to the 2.2 value reported previously [53]. As additional example, Figure 3 presents the growing curves of the STD (in percentages), as calculated from the spectra obtained at 290 K. They are represented as functions of the saturation time and the sugar concentration.

The percentage of saturation follows the standard definition in STD experiments, see equation 20 [45]:

%STD = 100 × (I*_off_*_res_ − I*_on_*_res_)/I*_off_*_res_ (20)

where I*_off_*_res_ corresponds to the signal intensity in the *off*-resonance spectrum, I*_on_*_res_ to the signal intensity in the *on*-resonance spectrum and (I*_off_*_res_ − I*_on_*_res_) to the signal intensity in the difference STD spectrum (Figure 3). This percentage of saturation for each anomer can easily be transformed into the formal concentration of each anomer already saturated by multiplying by the total anomer concentration, see methods, equations 18 and 19.

Then it is possible to apply a kinetic model to these saturation progress curves, similar to the treatment of an enzymatic reaction, where the steady-state saturated receptor during the *on*-resonance spectrum can be likened to an “enzyme” catalyzing the transfer of saturation to the ligand. The following chemical equilibrium and kinetic steps must be considered:

[R] = [R*] (concentration of receptor, whose spins are steady-state saturated (R*) in the *on*-resonance spectra).

Binding equilibrium:

R* + L ⇆ R*L

Kinetic step of saturation transfer:

R*L → R*L*

Dissociation and rebinding of already saturated ligand rendering an unproductive complex:

R* + L* ⇆ R*L*

Relaxation of saturated ligand when not associated to the receptor:

L* → L

With this simple kinetic model, it is possible to calculate corresponding dissociation and kinetic constants:

K_D_, dissociation constant of the binding equilibrium, equation 20:

K_D_ = *k_off_*/*k_on_* = [R*].[L]/[R*L] = [R*].[L*]/[R*L*] (20)

*k_m_* = magnetization transfer kinetic constant of the kinetic step R*L → R*L*.

*k_r_* = spin relaxation velocity constant for the process L* → L related with the inverse of the longitudinal relaxation time T_1_.

The build-up curves of saturated ligand at different concentrations can now be used to estimate the three parameters, K_D_, *k_m_* and *k_r_* by applying non-linear fitting procedures used in enzymatic studies, as implemented in DynaFit [51,52].

Of special note in the case of the anomeric mixture, both anomers must be simultaneously considered as reciprocal inhibitors: one inhibits the binding of the other and *vice versa* [37]. Moreover, the system cannot be studied with a simple competitive inhibition approach by varying inhibitor and ligand concentrations independently, because the concentrations of both anomers are correlated by the anomerization equilibrium and both simultaneously vary. This behavior has already been described for enzyme inhibition studies, when the competitive inhibitor was a contaminant of the substrate (ratio inhibitor/substrate constant) [54] and, with relevance to glycan recognition, in the case of the binding of anomers of *N*-acetylglucosamine to lysozyme [37]. Thus, in the case of a given mixture of anomers in equilibrium (α_total_⇆ β_total_, c_anomer_ = [α]_total_/[β]_total_), a double set of equilibrium and kinetic equations with their corresponding constants (see Methods, equations 1 to 19) must be considered.

**Figure 4:**
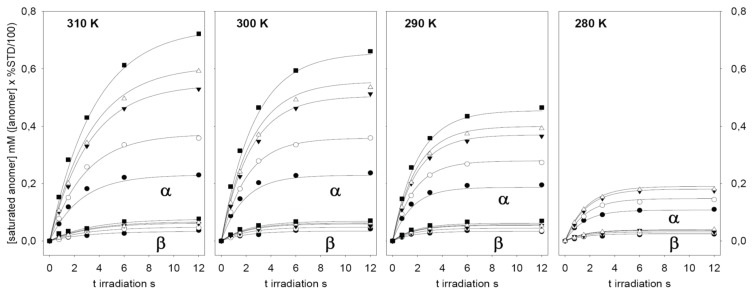
Time-dependent build-up curves of ligand saturation, expressed in mM, obtained for α-2FDM (upper lines) and β-2FDM (lower lines) in the presence of ConA (90 µM). Five different total concentrations of 2FDM, *i.e*., 0.96 (●); 1.94 (○); 4.14 (▼); 5.57 (Δ); and 12.1 mM (■), were studied from 0 to 12 s irradiation time and at four temperatures. The solid lines correspond to calculated curves for each 2FDM concentration obtained from the global fitting of the experimental data at each temperature to the kinetic model (see Methods and text) with DynaFit [51] (data for the highest concentration of 2FDM at 280K was excluded from the fitting). Values derived from the fitting for the dissociation constants are in Table 1.

Assuming that bound α- and β-2FDM ligands (and the protein) adopt closely related geometries in their bound states, then magnetization transfer towards the fluorine atom in both anomers is carried out in a similar manner. As an approximation, the magnetization transfer constant *k_m_* will have the same value for both anomers (*k_m_*_α_ = *k_m_*_β_ = *k_m_*), reflecting that the ^19^F atom is axial in both cases. In sharp contrast, the ^1^H → ^1^H STD of the well-resolved anomeric protons will be based on their different axial or equatorial orientations, with distinct saturation pathways. In our case, the two anomers interact with ConA, albeit with different affinities (see Figure 4).

The calculated saturated concentrations of both anomers from the experimental STD percentages at every concentration and saturation time were globally fitted to a series of kinetic differential equations corresponding to the above model using the DynaFit software [51] (Figure 4). Instead of directly using the equilibrium dissociation constants (K_Dα_ and K_Dβ_), the kinetic dissociation constants (*k_off_*_α_ and *k_off_*_β_) were used as adjustable parameters. First, the association constant *k_on_* was considered to be constant and fixed to the diffusion-limited encounter at 10^8^ M^−1^·s^−1^.This is a reasonable approximation of the kinetics of binding monosaccharides to ConA. Interestingly, it has been shown that, when analyzing STD data in processes that are not diffusion-controlled, the kinetics of the association must be considered [55]. Therefore, our kinetic model allows for checking the dependency of the dissociation constant by varying the association kinetics. Using this approach, it was possible to satisfactorily confirm that the use of *k_on_* values even as small as 10^5^ M^−1^ s^−1^ did not appreciably modify the obtained equilibrium dissociation constants. We can take 10^5^ as a safe lower limit considering the reported value measured by SPR for the association constant between ConA and mannan polisaccharides [56]. The relaxation constants (*k_r_*_α_ and *k_r_*_β_) for each anomer and the magnetization transfer constant, *k_m_* (the same for both anomers) were also taken as adjustable parameters (Table 1).

These relaxations velocities account for the observed flattening of the saturation build-up curves at long saturation times (Figure 3 and Figure 4). Interestingly, these relaxation velocities show a faster relaxation for the β anomer and also an inverse dependency on temperature: the observed values decreased when the temperature was increased. This behavior is also related with the inverse of the longitudinal relaxation time T1, experimentally measured for ^19^F in the absence of proton saturation (Table 1). Longer T1 values for the α anomer ^19^F signal were observed. Furthermore, the T1 relaxation times for both anomers increased with temperature, as expected for small molecules with faster molecular tumbling at high temperatures.

The data presented in Table 1 reveal a clear preference of ConA for the α anomer of 2FDM, in accordance with its known specificity for α-mannosides. It is possible to calculate an apparent macroscopic dissociation constant K_D_ from data obtained with the mixture of unsubstituted monosaccharides (Table 1). In this case, the combined dissociation constant for 2FDM (0.78 mM^−1^ at 290 K) is in the range for those described for mannose and glucose (0.45 mM^−1^ at 290 K and 1.8 mM^−1^ at 292 K, respectively) [32] or 2-fluorodeoxyglucose (2.2 mM^−1^ at 298 K) [33]. Interestingly, the observed anomer selectivity showed a temperature dependency. The preference towards the α anomer was increased from 2.2 times at 280 K to more than five times at 310 K.

**Table 1:** α- and β-2FDM dissociation (K_Dα_ and K_Dβ_), magnetization (*k_m_*) and relaxation constants (*k_r_*_α_ and *k_r_*_α_) calculated by fitting to differential kinetic equations with DynaFit [51]. Experimental relaxivities (R = 1/T_1_) and anomeric ratios were measured at each temperature by inversion recovery NMR experiments and 1D- ^19^F spectral information obtained from the ratio of the corresponding peak of each anomer, respectively. Errors correspond to the standard deviation for each parameter obtained after non-linear fitting. For comparison, the calculated combined macroscopic dissociation constant corresponding to the total monosaccharide concentration is included.

Temperature K	280	290	300	310
K_Dα_ (mM)	0.43 ± 0.04	0.59 ± 0.04	0.68 ± 0.05	0.88 ± 0.05
K_Dβ_ (mM)	1.15 ± 0.2	2.1 ± 0.24	3.1 ± 0.5	4.7 ± 0.7
K_D_ (mM) ª	0.54	0.78	0.94	1.15
*k_r_*_α_ (s^−1^)	0.57 ± 0.02	0.46 ± 0.01	0.35 ± 0.01	0.25 ± 0.01
1/T_1α_ (s^−1^)	0.84	0.66	0.50	0.42
*k_r_*_β_ (s^−1^)	0.60 ± 0.09	0.53 ± 0.07	0.45 ± 0.09	0.30 ± 0.06
1/T_1β_ (s^−1^)	0.95	0.79	0.59	0.50
*k_m_* (s^−1^)	1.6 ± 0.06	3.1 ± 0.07	3.4 ± 0.01	2.84 ± 0.07
%α %β	65.9% 34.1%	65.8% 34.2%	64.5% 35.5%	63.3% 36.7%

ª Macroscopic K_D_ for total concentration of monosaccharide: K_D_ = ([α] + [β])*[R]/([Rα] + [Rβ]) and can be deduced K_D_ = (1 + 1*c_anomer_)*(K_α_*K_β_)/(K_α_ + K_β_*c_anomer_).

## 4. Conclusions

A broad range of techniques have been employed to monitor ligand binding to lectins in solution (for an overview, please see Table 1 in [57]). We here extend our previously developed concept of using ^19^F-NMR spectroscopy by ^1^H → ^1^H STDre^19^F monitoring [42,43]. Using a common test system (ConA, 2FDM), at different concentrations and temperatures, anomer selectivity could be measured, without signal overlap and without the requirement for chemical modifications. Obviously, the sensor capacity of ^19^F is sufficient for obtaining quantitative data and can thus inspire further applications in advancing our understanding of structural aspects of lectin-carbohydrate interaction in solution, e.g., when working with human lectins such as the adhesion/growth-regulatory galectins [58].

## Abbreviations

2FDM	2-fluoro-2-deoxy- d-mannose
2α-FDM	2-fluoro-2-deoxy-α- d-mannose
2β-FDM	2-fluoro-2-deoxy-β- d-mannose
ConA	concanavalin A
STD	Saturation Transfer Difference
NMR	Nuclear Magnetic Resonance
^1^H → ^1^H STDre^19^F-NMR	H,H-Saturation Transfer Difference (STD) experiment with relay to ^19^F
